# Characterization of Factors Associated with Tissue Immunity, Cellular Activity and Angiogenesis in Children with Unilateral Cleft Lip and Palate Before and During Primary Dentition: A Comparative Cross-Sectional Study

**DOI:** 10.3390/jcm14144952

**Published:** 2025-07-12

**Authors:** Laura Ozola, Māra Pilmane

**Affiliations:** Institute of Anatomy and Anthropology, Riga Stradins University, Kronvalda Boulevard 9, LV-1010 Riga, Latvia

**Keywords:** vascular endothelial growth factor A, transforming growth factor beta1, NF-kappa B, heat-shock proteins, CD163, macrophages, cleft lip, cleft palate, primary dentition

## Abstract

**Introduction**: Unilateral cleft lip and palate (CLP) is a severe orofacial birth defect characterized by improper fusion of facial parts and disturbed orofacial functions. The defect manifests as a gap in the orofacial tissues that is accompanied by defective healing patterns and chronic inflammation. The immune system’s defense factors modulate immunity, inflammation, and healing. Angiogenesis factors control blood-vessel formation. Therefore, these factors are vital in the immunological assessment and understanding of CLP morphopathogenesis. The aim of the study is to assess the distribution of vascular endothelial growth factor (VEGF), transforming growth factor beta 1 (TGF- β1), the total macrophage population and the M2 subtype, heat-shock proteins (HSP) 60 and 70, and nuclear factor kappa B (NF-κB) p50 and p65 subtypes in the affected tissue of children with CLP before and during primary dentition. **Materials and Methods**: Tissue samples were obtained from 15 patients aged from 3 to 8 months during veloplastic surgery. Five controls were used for comparison of data. Immunohistochemistry, light microscopy, semi-quantitative evaluation (from 0 to ++++), and statistics (Mann–Whitney U test and Spearman’s rank correlation) were used to evaluate the data for statistically significant differences and correlations between the groups. **Results**: Epithelial tissues affected by CLP presented with statistically significant increases in levels of VEGF (*p* = 0.007), total macrophages (*p* = 0.007), HSP60 (*p* = 0.001), NF-κB p65 (*p* = 0.000), and p50 (*p* = 0.045), but with a decrease in M2 macrophages (*p* = 0.025). Blood vessels in CLP-affected tissues showed a statistically significant increase in levels of NF-κB p65 (*p* = 0.003) and a statistically significant decrease in M2 numbers (*p* = 0.014). Connective tissue presented with no statistically significant differences. Spearman’s rank correlation revealed multiple statistically significant correlations—26 positive and 5 negative. **Conclusions**: Statistically significant changes in levels of VEGF and both NF-κB subtypes and numbers of total macrophages and M2 macrophages suggest a possible alteration of variable immune and inflammatory reactions and macrophage functions associated with the initiation and maintenance of the chronic process and the resulting damage.

## 1. Introduction

Unilateral cleft lip and palate is a severe orofacial defect that affects the nasal and oral region due to improper fusion of facial processes [1]. The severity of the congenital defect impairs the child’s functions of speech, nutrition, breathing, and proper orofacial and dental development.

In order to regain normal function of the orofacial region, children with unilateral cleft lip undergo a series of plastic surgeries starting at the age of 3 months. Due to the severity, characteristics, and location of the defect, complications often occur after the operation, with the most common ones being asymmetry, scarring, reopening of the wound, infection, and improper healing [1]. Possible causes of complications include the presence of chronic inflammation in clefted tissues; morphologic, functional, and molecular changes in the tissues; and decreased potential for proper tissue healing and remodeling [2]. Thus, indices of angiogenetic growth, cellular activity, and inflammation can be used to assess tissue homeostasis in the case of cleft lip and palate.

Vascular endothelial growth factor (VEGF) is a protein that functions as an endothelial specific growth factor and regulates angiogenesis and endochondral ossification [3]. The factor has also been noted to inhibit endothelial apoptotic signals, thus protecting and maintaining immature vasculature [4].

Transforming growth factor-beta 1 (TGF-β1) is a cytokine that functions to resolve inflammation and initiate tissue repair through regulation of cell proliferation, differentiation, and ECM production [5,6,7]. Additional functions of angiogenesis reduction and initiation of endothelial-cell apoptosis have also been observed [8].

Nuclear factor-kappa B (NF-kB) is a transcription factor that, through the regulation of transcription, the cell cycle, and various human genes, is an important mediator of inflammation, proliferation, differentiation, the immune response, cell growth, adhesion, and apoptosis resistance [9,10,11].

Heat shock proteins (HSP) are proteins that monitor the quality, structure, and function of other proteins, and thus ensure homeostasis of cellular proteins.

Macrophages are tissue-specific immune cells that ensure phagocytosis, initiate inflammation in the innate immune response, maintain tissue homeostasis, and sometimes participate in tissue repair and the resolution of inflammation [12]. The balance of pro-inflammatory activity and inflammation resolution is ensured through polarization of macrophages into the M1 (killer) and M2 (healer) phenotypes [13,14]. CD163 has been characterized as an important marker for M2 anti-inflammatory macrophages and their activation due to its high specificity to this cell type [15].

Tissue-associated immune factors are critical in mediating inflammation, immune responses, and tissue healing, and previous studies have sought to connect them to the development of certain oral and maxillofacial pathologies. Angiogenesis factors are crucial in the formation of new blood vessels and the resolution of inflammation. Both tissue-associated immune factors and angiogenesis factors have been proven to serve as synergistic links in chronic inflammation processes due to their similar triggers, induction mechanisms, and homeostatic properties [16].

Despite previous research, it is still unclear how cellular activity, tissue homeostasis, and vascular processes change in cleft-affected tissues themselves and how these changes vary across age groups. Different tissue factors like interleukins, human beta defensins and cathelicidins have been previously studied only in older patients—during mixed or permanent dentition. The research data on patients during primary dentition are scarce. Moreover, the combination of tissue-associated immune and angiogenesis factors, which is the focus of this particular study, has not been researched ever before, despite the fact that these factors regulate important processes in the orofacial region.

Previous research, mostly that conducted by the research group Pilmane et.al, has shown that cleft-affected tissues present with a significant decrease in levels of various growth factors such as TGF-β3; a decrease in levels of apoptotic markers; an increase in levels of various inflammatory and immune markers (NF-κB, pro-inflammatory interleukins, TNF-α, LL-37 and others); a decrease in levels of vascular, angiogenic, and remodeling markers (VEGF, matrix metalloproteinases); and changes in several genes and levels of their encoded proteins, such as bone morphogenetic protein 2/4, SRY-related HMG-box 2, and forkhead box O1 [17,18,19].

For these reasons, further research of tissue-associated immune and angiogenesis factors and their possible interplay will contribute to an increased understanding of molecular morphopathological mechanisms underlying inflammatory diseases and the development of novel therapeutic approaches for oral and maxillofacial pathologies.

Since clefted tissue is characterized by chronic inflammation and compromised healing, the aim of this study is to assess and compare the distribution of tissue-associated immune and angiogenesis factors in the tissues associated with unilateral cleft lip and palate in children during primary dentition. The results of this research will continue an ongoing cycle of research aiming to discover new possible links between tissue factors and the characteristics of clefted tissue.

## 2. Materials and Methods

### 2.1. Ethical Approvals

This research was conducted in accordance with the 1975 Helsinki Declaration (as revised in 2008). The study was independently reviewed and approved by the Ethical Committee of the Riga Stradins University (22 May 2003; 17 January 2013; Nr. 5/28 June 2018).

All parents of the patients were fully informed about the nature of this study, and they provided written informed consent for participation in the study and its publication.

The ethical committee approval Nr. 2-PEK-4/595/2022 for the use of the control group’s tissues was issued on 14 December 2022.

### 2.2. Selection Criteria of Patient Tissue Samples

Patients were selected according to the following inclusion criteria:

- Diagnosis of unilateral cheilognathouranoschisis (cleft lip and palate);
- Age before and during primary dentition of birth–5 years;
- Absence of other congenital pathologies;
- No symptoms of active and acute inflammation;
- Absence of additional pathologies that contraindicate surgery;
- Indications for plastic surgery for cleft repair.

Patients were excluded if any of the following criteria applied:

- Presence of other congenital pathologies;
- Symptoms of active and acute inflammation;
- Contraindicative pathologies for cleft-repair surgery;
- Age during mixed dentition of 6–12 years or presence of permanent dentition.

### 2.3. Description of Selected Patients

In total, 15 patients were selected, and from these patients, 15 samples of lip tissue were obtained during cheiloplastic surgery in the Cleft Lip and Palate Centre of the Institute of Stomatology of Riga Stradins University. The patient age varied from three to eight months old. Nine of the selected patients were male, and six were female.

Two of the patients had family histories of clefts; one patient had a mother who smoked during pregnancy; one patient had a family history of Down’s syndrome; four patients had parents with other chronic/acute illnesses of other organ systems; and seven patients had no previous history of illness in their family anamnesis. All patients underwent cheiloplasty. Information about each patient’s age, sex, and family anamnesis is summarized in Table 1.

### 2.4. Selection Criteria for Control Tissue Samples

Control samples were selected according to the following criteria:

- Absence of craniofacial cleft on patient examination, anamnesis, and family history;
- Absence of subsidiary pathologies and hereditary anomalies;
- No damage to the tissues of the oral cavity; no presence of inflammation.

### 2.5. Description of Control Group

For the control group, in total, five tissue samples were acquired from the Institute of Anatomy and Anthropology of Riga Stradins University during post-mortem necropsies.

Four of the selected controls were female, and one was male. The control age varied from newborn to 24 weeks old. For two of the controls, the cause of death was birth asphyxia; for two, it was sudden infant death syndrome; and for one, it was abortion due to maternal health status (Table 2).

### 2.6. Routine Staining

The routine staining of all samples was performed according to the following steps: fixation for 24 h with 2% formaldehyde, 0.2% picric acid, and 0.1 M phosphate buffer (pH 7.2); processing for 12 h with Tyrode’s buffer with 10% saccharose; embedding of the tissues in paraffin; cutting with a microtome into 5–7 µm sections; and staining with hematoxylin and eosin [21].

### 2.7. Immunohistochemical (IHC) Analysis

Immunohistochemical quantification of tissue-associated immune and angiogenesis factors in the tissue samples was performed using the standard streptavidin and biotin method [22,23]. Firstly, dilution of antibodies in antibody diluent was performed (code-938B-05, Cell MarqueTM, Rocklin, CA, USA).

This step was followed by the preparation of tissue samples for antibodies, which was carried out as follows: deparaffinization of previously cut tissue samples, washing of samples in alcohol and water, rinsing with tris buffer solution (code-2017X12508, Diapath S.p.A., Martinengo, Italy) two times for 5 min each, placing of samples in a microwave with boiling EDTA buffer (code-2017X02239, Diapath S.p.A., Martinengo, Italy) for 20 min and cooling, washing with tris buffer 2 × 5 min, blocking with 3% peroxide for 10 min, and washing with tris buffer.

The antibody reaction was performed as follows: incubation of samples with primary antibodies for 1 h, washing of samples with tris buffer three times, exposing the samples to the HiDef DetectionTM reaction amplifier (code 954D-31, Cell MarqueTM, Rocklin, CA, USA) for 10 min at room temperature, and washing with tris buffer three times for 5 min each.

The information about the antibodies used is summarized in Table 3.

Lastly, the samples were prepared for sealing as follows: tissue coating with DAB+ chromogenic liquid DAB Substrate Kit (code 957D-60, Cell MarqueTM, Rocklin, CA, USA) for 10 min, rinsing with running water, counterstaining with hematoxylin (code-05-M06002, Mayer’s Hematoxylin, Bio Optica Milano S.p.A., Milano, Italy), dehydration with ethanol of increasing concentrations (70°–90°), clarification with carboxylic acid and xylol, sealing with a coverslip, and marking according to the patient number and antibody used. The marking of samples was combined with blinding—each of the patient and control samples was assigned a number, which was used for each of the factors. After evaluation of each slide, the numbers were then decoded and the samples and results were organized into patient and control groups.

### 2.8. Evaluation of Factor Quantity

Light microscopy and semi-quantitative evaluation were used to assess the relative quantities of VEGF, TGF-β1, NF-κB p105/p50, NF-κB p65, HSP 60, HSP 70, macrophages, and CD163-positive structures in the epithelium, connective tissue, and blood vessels. Evaluation of positively-stained structures was performed according to identifiers summarized in Table 4. Evaluation was performed by two separate raters, and the results were then compared. Due to findings of almost identical results between the raters, additional inter-rater and intra-rater tests were not performed.

Acquisition, processing, and analysis of pictures of tissue samples were performed using a Leica DC 300F digital camera (Leica Microsystems Digital Imaging, Cambridge, UK) and the Image Pro Plus 5.0 program (Media Cybernetics, Inc., Rockville, MD, USA).

### 2.9. Statistical Analysis

IBM SPSS (Statistical Package for the Social Sciences) software version 26.0 (IBM Company, Chicago, IL, USA) was used for statistical processing of the data. Statistical significance was selected at a *p*-value < 0.05 and was used for every statistical assessment of the tests and results [26]. Semi-quantitative evaluation of the amounts of defense factors in the tissue produced ordinal data (i.e., data that were non-numeric and arranged in a specific and unchangeable order); therefore, descriptive statistics, analytical statistics, and non-parametric tests were used to calculate the results and their statistical significance, as described below.

#### 2.9.1. Mann–Whitney U Test

This test was used to detect differences between the distributions of factor quantities in samples from the patient and control groups and determine their statistical significance [26].

#### 2.9.2. Spearman’s Rank Correlation

This test was used to detect statistically significant correlations between changes in one factor and changes in another factor [26]. The strength of correlation between factors was interpreted using the following definitions of Spearman’s rho (r_s_) values:

- a very weak correlation: r_s_ = 0.00–0.19;
- a weak correlation: r_s_ = 0.20–0.39;
- a moderate correlation: r_s_ = 0.40–0.59;
- a strong correlation: r_s_ = 0.60–0.79;
- a very strong correlation: r_s_ = 0.80–1.00 [27].

#### 2.9.3. Data Visualization

The results obtained via semi-quantitative evaluation, the Mann–Whitney U test and Spearman’s rank correlation were processed into graphs and a correlation matrix using Microsoft Excel (2024).

### 2.10. Summary Flowchart

**Figure 1:**
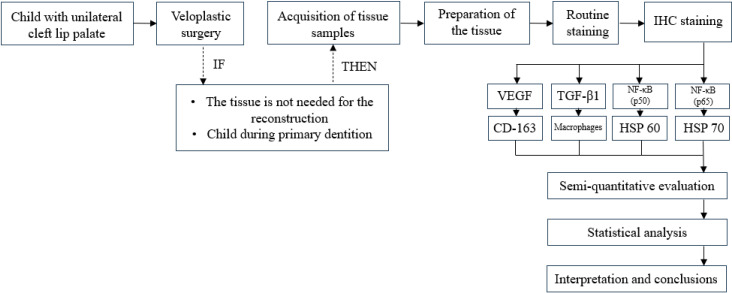
Workflow of selection of patient tissue samples, processing of samples, and research. Abbreviations: VEGF, vascular endothelial growth factor; TGF-β1, transforming growth factor beta 1; NF-κB, nuclear factor kappa B; HSP, heat-shock protein.

A visual summary of the workflow and information presented in the Section 2 is outlined in Figure 1.

## 3. Results

### 3.1. Description of Routine Staining

**Figure 2:**
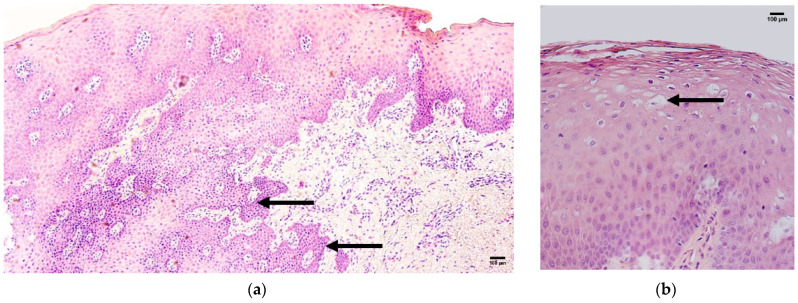
Routine hematoxylin-and-eosin staining of the patient tissue samples. (**a**) Web-like epithelial ingrowth and infiltration of inflammatory cells (arrows), magnification 100×; (**b**) and vacuolization (arrows), magnification 200×.

The control samples showed normal features of lip tissue—a mucosal part with non-keratinized stratified squamous epithelium and lamina propria and a skin part with keratinized stratified squamous epithelium containing sweat glands, ducts, hair follicles, and lamina propria. The patient tissue samples presented with prominent and web-like ingrowth of epithelial tissue into the underlying mucosal connective tissue and subepithelial infiltration of inflammatory cells (Figure 2a), as well as prominent epithelial vacuolization (Figure 2b).

### 3.2. Appearance and Distribution of VEGF

**Figure 3:**
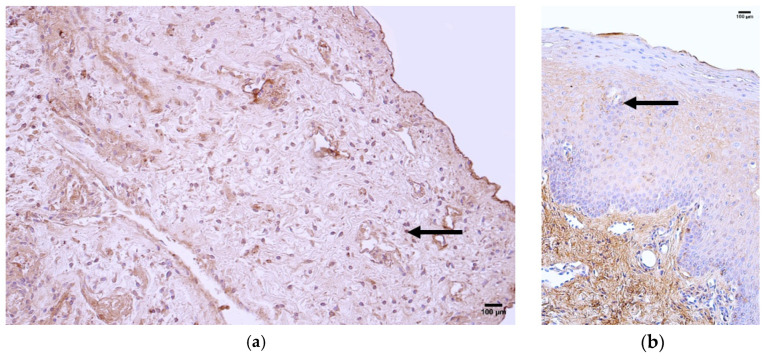
Immunohistochemistry of the VEGF-positive structures in the control and patient tissue samples. (**a**) Control sample with a moderate number of positive structures in the connective tissue (arrows) and few in the blood vessels (arrows), 200×; (**b**) patient sample with a moderate number of positive structures in the epithelium and few positive structures in the connective tissue and blood vessels (arrows), 200×.

The control group presented a median of zero (0) VEGF-positive structures in the epithelium and few (+) positive structures in the connective tissue and blood vessels (Figure 3a, Table 5). 

The patient group presented with median values of moderate to numerous (++) VEGF-positive structures in the epithelium, rare (0/+) positive structures in the connective tissue, and few (+) positive structures in the blood vessels (Figure 3b, Table 5).

Comparison of the patient and control groups using the Mann–Whitney U test showed a statistically significant difference in the epithelium (U = 7.307 *p* = 0.007) (Table 6). Additionally, no statistically significant differences were observed in the connective tissue (U = 2.722, *p* = 0.099) or blood vessels (U = 0.887, *p* = 0.346) (Table 6).

### 3.3. Appearance and Distribution of TGF-β1

**Figure 4:**
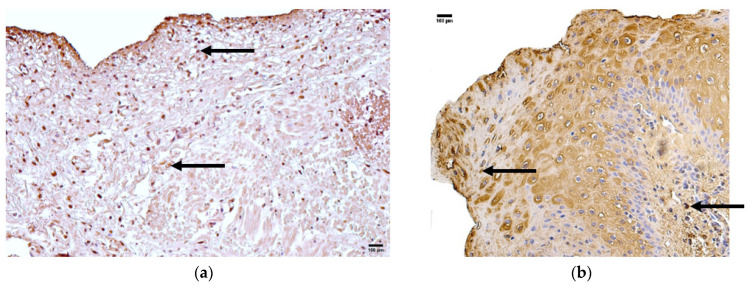
Immunohistochemistry of the TGF-β1 positive structures in the control and patient tissue samples. (**a**) Control sample with few positive structures in the connective tissue and blood vessels (arrows), 200×; (**b**) patient sample with few positive structures in the epithelium, few to moderate numbers of positive structures in the connective tissue, and few positive structures in the blood vessels (arrows), 200×.

The control group presented median values of few (+) TGF-β1 positive structures in the epithelium, connective tissue, and blood vessels (Figure 4a, Table 5). 

The patient group presented with median values of few (+) TGF-β1 positive structures in the epithelium and blood vessels but few to moderate (+/++) numbers of positive structures in connective tissue (Figure 4b, Table 5).

Comparison of the patient and control groups using the Mann–Whitney U test showed no statistically significant differences in the epithelium (U = 0.019, *p* = 0.0889), connective tissue (U = 0.101, *p* = 0.750), or blood vessels (U = 3.004, *p* = 0.083) (Table 6).

### 3.4. Appearance and Distribution of NF-κB p105/p50

**Figure 5:**
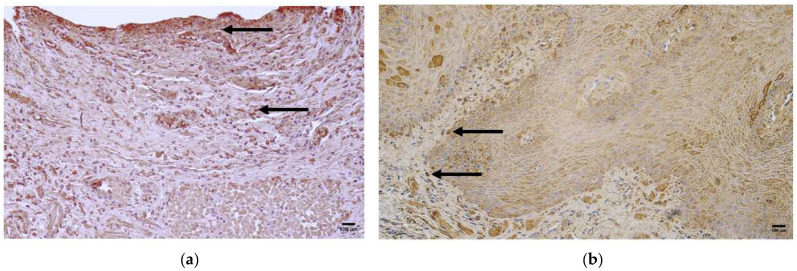
Immunohistochemistry of the NF-κB p105/p50-positive structures in the control and patient tissue samples. (**a**) Control sample with numerous positive structures in the connective tissue and blood vessels (arrows), 200×; (**b**) patient sample with moderate to numerous positive structures in the epithelium, few to moderate numbers in the connective tissue, and few positive structures in the blood vessels (arrows), 200×.

The control group presented with median values of few (+) NF-κB p105/p50-positive structures in the epithelium and moderate (++) numbers of positive structures in the connective tissue and blood vessels (Figure 5a, Table 5). 

The patient group presented with median values of moderate to numerous (++/+++) NF-κB p105/p50-positive structures in the epithelium, few to moderate numbers (+/++) of positive structures in the connective tissue, and moderate (++) numbers of positive structures in the blood vessels (Figure 5b, Table 5).

Comparison of the patient and control groups using the Mann–Whitney U test showed a statistically significant difference in the epithelium (U = 4.004, *p* = 0.045) (Table 6). Additionally, no statistically significant differences were observed in the connective tissue (U = 2.156, *p* = 0.142) or blood vessels (U = 1.267, *p* = 0.260) (Table 6).

### 3.5. Appearance and Distribution of NF-κB p65

**Figure 6:**
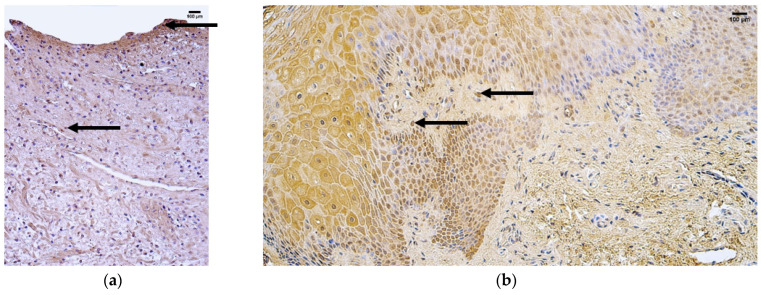
Immunohistochemistry of the NF-κB p65-positive structures in the control and patient tissue samples. (**a**) Control sample with few to moderate positivenumbers of positive structures in connective tissue and few in blood vessels (arrows), 200×; (**b**) patient sample with numerous positive structures in the epithelium, few positive structures in the connective tissue, and numerous positive structures in the blood vessels (arrows), 200×.

The control group presented with median quantities of moderate to numerous NF-κB p65-positive structures in the epithelium, few positive structures in the connective tissue and moderate to numerous positive structures in the blood vessels (Figure 6a, Table 5). 

The patient group presented with the median quantity of numerous NF-κB p65-positive structures in the epithelium, few positive structures in the connective tissue, and moderate to numerous positive structures in blood vessels (Figure 6b, Table 5).

Comparison of the patient and control group using the Mann–Whitney U test showed a statistically significant difference in the epithelium (U = 12.873, *p* = 0.000) and in blood vessels (U = 8.842, *p* = 0.003) (Table 6). Additionally, no statistically significant differences were observed in the connective tissue (U = 0.051, *p* = 0.821) (Table 6).

### 3.6. Appearance and Distribution of HSP 60

**Figure 7:**
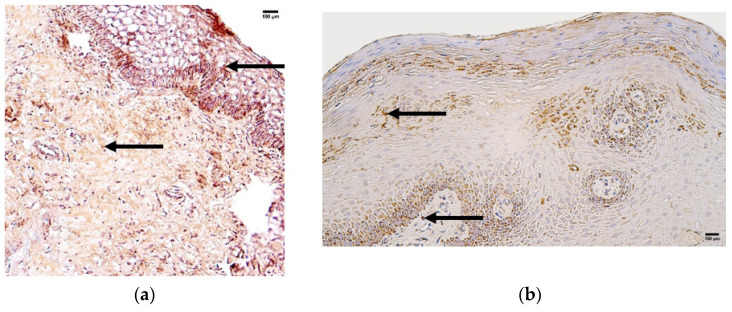
Immunohistochemistry of the HSP 60-positive structures in the control and patient tissue samples. (**a**) Control sample with few positive structures in the epithelium and connective tissue and no positive structures in blood vessels (arrows), 200×; (**b**) patient sample with a moderate number of positive structures in the epithelium and few in the connective tissue and blood vessels (arrows), 200×.

The control group presented with median quantities of no (0) HSP 60-positive structures in the epithelium, connective tissue, and blood vessels (Figure 7a, Table 7). 

The patient group presented with a median quantity of moderate to numerous (++/+++) HSP 60-positive structures in the epithelium and rare (0/+) positive structures in connective tissue and blood vessels (Figure 7b, Table 7).

Comparison of the patient and control groups using the Mann–Whitney U test showed a statistically significant difference in the epithelium (U = 11.229, *p* = 0.001) (Table 8). Additionally, no statistically significant differences were observed in connective tissue (U = 0.693, *p* = 0.405) and blood vessels (U = 1.867, *p* = 0.172) (Table 8).

### 3.7. Appearance and Distribution of HSP 70

**Figure 8:**
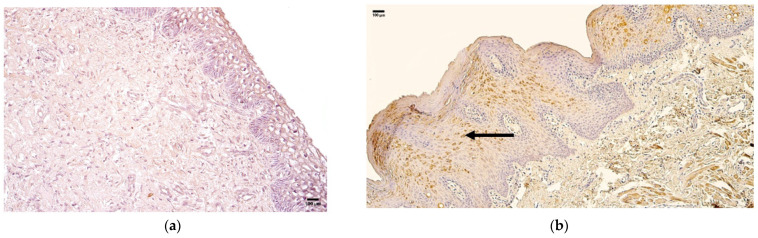
Immunohistochemistry of the HSP 70 positive structures in the control and patient tissue samples. (**a**) Control sample with no positive structures in the epithelium, connective tissue, or blood vessels. 200×; (**b**) patient sample with few to moderate numbers of positive structures in the epithelium and no positive structures in the connective tissue or blood vessels (arrows), 200×.

The control group presented with a median quantity of no (0) HSP 70 positive structures in the epithelium, connective tissue, or blood vessels (Figure 8a, Table 7). 

The patient group presented with a median quantity of few to moderate (+/++) HSP 70 positive structures in the epithelium and no (0) positive structures in connective tissue and blood vessels (Figure 8b, Table 7).

Comparison of the patient and control groups using the Mann–Whitney U test showed a statistically significant difference in the epithelium (U = 11.301, *p* = 0.001.) (Table 8). Additionally, no statistically significant differences were observed in the connective tissue (U = 0.667, *p* = 0.414) or blood vessels (U = 0.040, *p* = 0.841) (Table 8).

### 3.8. Appearance and Distribution of Macrophages

**Figure 9:**
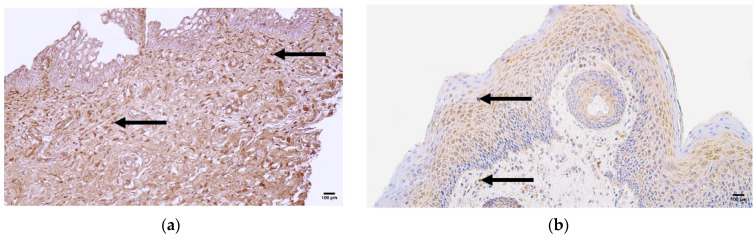
Immunohistochemistry of the macrophages in the control and patient tissue samples. (**a**) Control sample with a moderate number of positive structures in connective tissue (arrows), 200×; (**b**) patient sample with numerous positive structures in the epithelium and few in the connective tissue (arrows), 200×.

The control group presented with a median quantity of few (+) macrophages in the epithelium, connective tissue, and blood vessels (Figure 9a, Table 7). 

The patient group presented with a median quantity of numerous (+++) macrophages in the epithelium, few (+) positive structures in the connective tissue, and moderate (++) positive structures in the blood vessels (Figure 9b, Table 7).

Comparison of the patient and control groups using the Mann–Whitney U test showed a statistically significant difference in the epithelium (U = 7.331, *p* = 0.007) (Table 8). Additionally, no statistically significant difference was observed in the connective tissue (U = 0.307, *p* = 0.579) or blood vessels (U = 0, *p =* 1) (Figure 7a, Table 8).

### 3.9. Appearance and Distribution of M2 Macrophages

**Figure 10:**
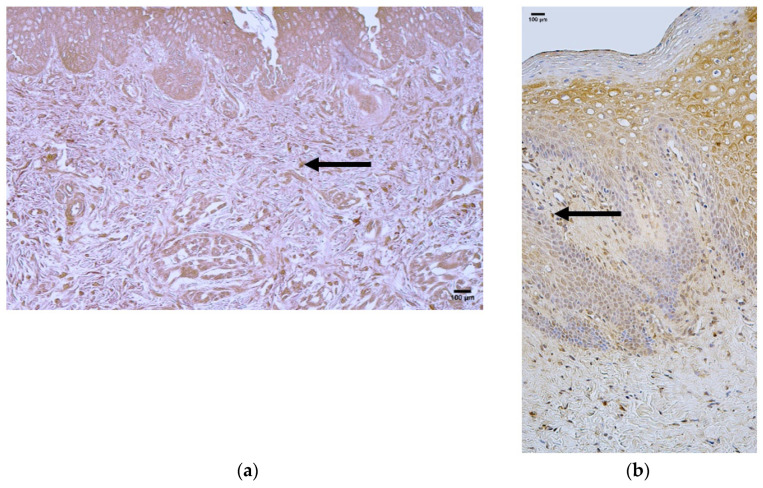
Immunohistochemistry of the CD163-positive structures in the control and patient tissue samples. (**a**) Control sample with no positive structures in the epithelium and few in connective tissue (arrows), 200×; (**b**) patient sample with no positive structures in the epithelium and moderate in connective tissue (arrows), 200×.

The control group presented with a median quantity of few (+) CD163-positive structures in the epithelium, connective tissue, and blood vessels (Figure 10a, Table 7). 

The patient group presented with median values of no (0) CD163-positive structures in the epithelium, few (+) positive structures in connective tissue and rare (0/+) positive structures in blood vessels (Figure 10b, Table 7).

Comparison of the patient and control groups using the Mann–Whitney U test showed a statistically significant difference in the epithelium (U = 5.030, *p* = 0.025) and in the blood vessels (U = 6.095, *p* = 0.014) (Table 8). Additionally, no statistically significant differences were observed in the connective tissue (U = 1.335, *p* = 0.248) (Table 8).

### 3.10. Comparison of Appearance and Distribution of Defense Factors

**Figure 11:**
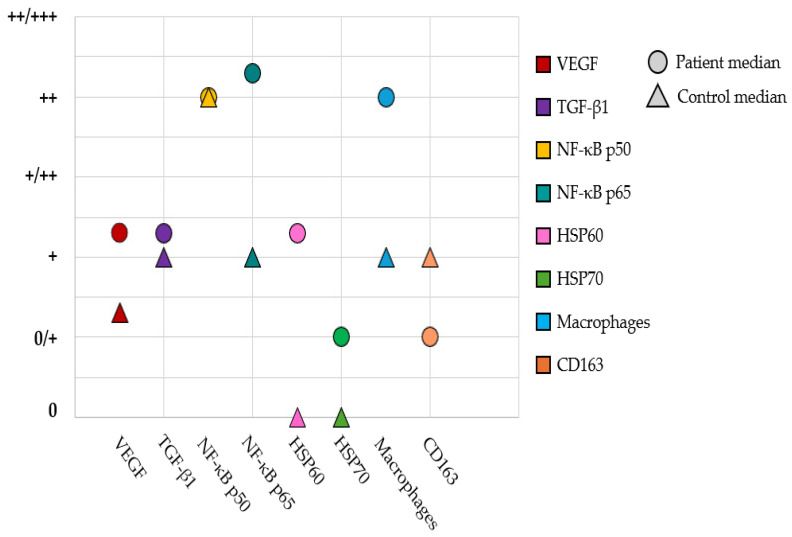
Comparison of distributions of median values for factors in patient and control-group tissues. Abbreviations: VEGF, vascular endothelial growth factor; TGF-β1,transforming growth factor beta 1; NF-κB, nuclear factor kappa B; HSP, heat-shock protein.

A visual summary of the comparison between median values for factors in all tissues in the patient and control group samples is given in Figure 11. Each factor has been marked with its own color, and the patient-group median has been marked with a circle, while median value for the control group is marked with a triangle. The most notable differences in factor distribution can be seen in NF-κB p65, HSP60, and macrophages.

### 3.11. Statistically Significant Correlations Between the Factors in Tissues of the Patient Group

**Figure 12:**
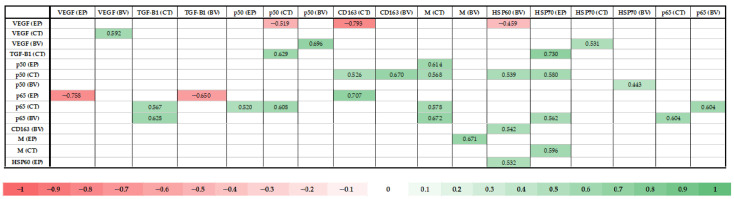
Correlation matrix of statistically significant correlations between tissue-associated immune and angiogenesis factors. Abbreviations: VEGF, vascular endothelial growth factor; TGF-β1, transforming growth factor beta 1; NF-κB, nuclear factor kappa B; HSP, heat-shock protein.

Using Spearman’s rank correlation coefficient, multiple statistically significant correlations (*p* < 0.005) were found between the factors in the epithelium, connective tissue and blood vessels. The factors presented 16 positive correlations (green) and 3 negative correlations (red), with the greatest number of statistically significant correlations being present in blood vessels (Figure 12).

## 4. Discussion

In our study, the epithelium showed statistically significant increase in the levels of all factors except CD163 and TGF-β1. Blood vessels showed a significant decrease in the number of CD163+ M2 macrophages.

### 4.1. VEGF

VEGF functions as an endothelial specific growth factor and regulates angiogenesis (expansion of the vascular network) and endochondral ossification (formation of bones from the cartilage model) [3]. Due to stimulation of blood-vessel growth and blood flow, VEGF also can modulate the immune response, wound healing, and scar formation [28]. In our study, VEGF levels were increased in the epithelium; therefore, it can be suggested that the increase in the levels of this factor could indicate hypertrophic scarring of the tissue [28]. Another possible cause for VEGF elevation can be explained by hypoxia serving as an expression-inducing factor [3,4]. This could be specifically relevant when looking at tissues associated with cleft lip and palate, as the veloplastic surgery creates trauma that can promote tissue hypoxia. Increase in VEGF levels after the trauma of surgery and hypoxia may be a pivotal mechanism that explains the excessive scarring seen in surgically corrected cleft defects.

### 4.2. TGF- β1

TGF- β1 is an important connective-tissue cytokine that functions to resolve inflammation and initiate tissue repair through activation of fibroblasts or inhibition of most other cells [5,6]. Increased TGF- β1 expression is connected to scarring and fibrosis of healing tissues [6,8]. Overexpression of this factor has been noted in fibrotic healing in inflammatory airway diseases, oral submucosal fibrosis, and oral squamous cell carcinoma [5,8,29]. TGF-β1 is also an important promoter of correct fusion of the palatal shelves [7]. Previous research has shown that in the case of improper palatal shelve fusion and cleft lip and/or palate pathology, fibroblasts are defective and produce significantly less TGF- β1 [7]. However, in our study, TGF- β1 showed no statistically significant changes, indicating a lack of connective-tissue involvement in the morphopathogenesis of cleft lip and palate.

### 4.3. MACROPHAGES and CD163

Macrophages are tissue-specific immune cells with a wide variety of immunological, inflammatory, and homeostatic functions [12]. Macrophages, based on the type of activation cascade they undergo, can polarize into two subtypes: M1 (killer, pro-inflammatory) and M2 (healer, anti-inflammatory) [13,14,15,30]. Polarization of macrophages into phenotypes creates a delicate equilibrium between pro- and anti-inflammatory occurrences in the body and ensures balance between inflammation and its resolution [13,14].

CD163 is a distinct receptor expressed by macrophages that therefore is used as an important marker for M2 anti-inflammatory macrophages and their activation [15,31]. The main functions of CD163 and M2 macrophages include secretion of IL-10, nitric oxide (NO), and IL-6; improved wound healing and angiogenesis; inhibition of T-cells; downregulation of inflammation; and even promotion of tissue remodeling [32,33,34,35]. Moreover, a connection has also been made between extent of fibrosis and CD163 expression, suggesting a role for M2 macrophages in the production of connective tissue as a protective response against prolonged inflammation [36].

This study found a significant increase in total macrophage numbers in the epithelium and significant decrease in the amount of CD163 in the epithelium and blood vessels. This phenomenon could illustrate a possible shift in macrophage equilibrium towards pro-inflammatory M1 macrophages and inflammatory processes. The shift can be explained by the anatomical features of the defect—the constant connection to the outer environment and the oral environment ensures the easy passage of bacteria from these sources into the tissues of the cleft lip and palate.

However, previous research on cleft-affected palate tissue has found no significant changes in CD163 levels [37]. Therefore, these results indicate the need for more research in order to precisely analyze the changes in M2 macrophage subtypes and the possible causes and effects of those changes.

### 4.4. HSP 60

Heat-shock proteins monitor the quality, structure, and function of other proteins and thus ensure homeostasis of cellular proteins [38,39]. HSP60 is mainly found intracellularly, in the mitochondria, where it ensures functions of the mitochondrial respiratory chain and cell survival [38,39]. Cytosolic HSP60 can also modulate the expression of NF-kB and protect cells from oxidative stress [39]. HSP60 expression has been noted to increase in inflammatory conditions [39]. In inflammatory diseases, HSP60 triggers cytokine release, NF-kB activation, oxidative stress, and the secretion of the pro-inflammatory cytokines IL-4 and IL-10 [39]. In this study, HSP60 presented a statistically significant increase in the epithelium. This could be possibly explained by the fact that cleft lip and palate is characterized by chronic inflammation, where the active functions of immune cells release ROS and create oxidative stress conditions; HSP60 is therefore overexpressed in order to protect the epithelial cells from further collateral damage driven by the chronic inflammation [39]. Moreover, the overexpression of HSP60 could be linked to promotion of tissue regeneration, as has been previously described by Pei et al. [40].

### 4.5. HSP70

HSP70 is a protein whose main purpose is to protect the cell against severe cellular stress, which could be thermic, chemical, bacterial, metabolic, or hypoxic [41,42]. In the immune response, HSP70 plays a dual role—it activates NK cells and inflammatory cell lysis and it also activates T regulatory cells, which promote upregulation of IL-10 and TGF-B and downregulation of IFN-γ and TNF-α [43]. The immunosuppressive activity of HSP70 is also expressed through the activation of B regulatory cells, which enhances their regulatory activities—decreasing pathological immunological responses, reducing uncontrolled and chronic inflammation, and suppressing the course of autoimmune disease [33]. A hypertonic environment has also been proven to be an initiator for increased HSP70 expression [44]. This finding is connected to the oral epithelium and its natural capacity to protect itself from hypertonic and osmolar stress [44]. In this study, HSP70 levels showed a significant increase in the epithelium. This difference could possibly be explained by osmolarity changes that induced HSP70 expression [44]. Since cleft lip and palate is characterized by chronic inflammation, the environment is most likely to be hypertonic due to high concentrations of immune cells, cytokines, metabolites, ions, and tissue/cell remains. This environment can act as a signaling activator, inducing amplified HSP70 expression, where HSP70 acts to suppress the active inflammation and protect the oral epithelium from hypertonic and osmolar stress.

### 4.6. NF-κB p50 and p65

Nuclear factor-kappa B (NF-κB) is a transcription factor that, through regulation of transcription, the cell cycle, and various human genes, is an important mediator of inflammation, proliferation, differentiation, the immune response, and apoptosis resistance [9,10,11]. NF-κB has also been noted as a possible driver of chronic inflammation [10,45]. Its expression can be stimulated by tobacco smoking, viruses, germs, radiation, stress signals, free radicals, endotoxins, proinflammatory cytokines, lymphocytes, and pH extremes [9]. One of the factor’s activation mechanisms (atypical) is induced by hypoxia and reactive oxygen species [10]. The NF-κB family consists of five members; p50 is synthesized as inactive protein, but p65 is synthesized with a transcriptional activation domain [9,10]. p50 functions as a regulator of transcription in two ways—firstly, by co-operating with p65, which ensures expression of pro-inflammatory genes, and, secondly, by acting as a homodimer and repressing transcription [10]. In this study, levels of both p50 and p65 were significantly increased. These changes could be explained by the presence of chronic inflammation, tissue hypoxia, and oxidative stress. However, the link between NF-κB and inflammatory processes can also reflect causation in the opposite direction—the initial increase in levels of NF-κB and thus in its transcriptional activity could possibly be the main driver of the creation and maintenance of chronic inflammation in the clefted epithelium.

### 4.7. Correlations

This study revealed multiple statistically significant pairwise correlations—positive and negative—for all of the factors. The presence of correlations does not always indicate causality; therefore, the interpretation of these results is speculative:

**VEGF.** Negative correlations with NF-κB p50 and p65, CD163, and HSP60. The negative correlation with HSP60 is contrary to results previously published in the literature, where VEGF and HSP60 were noted to be induced by the same stimulus—hypoxia [3,4,39]. In addition, the correlation with CD163 is also contrary to results in the existing literature, where macrophages have been found to initiate release of VEGF [46]. The correlations with both NF-κB subtypes contradict the existing literature, where both factors are described as having the same activating stimulus—hypoxia. The literature also describes NF-kB’s ability to directly upregulate VEGF transcription and a related positive feedback loop, with VEGF increasing NF-kB activity after angiogenesis [3,4,10,47]. Possible reasons for this contradiction could be the interplay of other signaling pathways (STAT3 or MAPK), the creation of cell- or tissue-specific negative feedback loops, or the severity of hypoxia as a modifying factor.

**TGF-β1.** Positive correlations with NF-κB p50 and p65, HSP70. These results suggest that contribution of connective-tissue involvement to the morphopathogenesis of cleft lip and palate is not direct, but, more likely, indirect, with close cooperation between the transcription and protein-regulatory factors and amplification of their functions.

**NF-κB p50.** Positive correlations with CD163, macrophages, HSP60 and 70 and NF-κB p65. Correlations with HSP indicate the ability of HSP to activate NF- κB, as was described by Singh et.al [39]. Correlations with CD163 and macrophages indicate the existence of an NF-κB-activation pathway through macrophage TLR recognition, as described by Zinatizdeh [11]. Lastly, the correlation with the p65 subtype indicates the close co-operation of both NF-κB family members [10].

**CD163.** The positive correlation with HSP60 illustrates a possible mechanism by which HSP60 may induce macrophage polarization into the M2 subtype [40].

**Macrophages.** The positive correlation with HSP70 possibly indicates the pro-inflammatory nature of extra-cellular HSP70, which promotes macrophage activation and maturation and cytokine release [48]. Moreover, HSP70 has been noted to be a possible initiator for macrophage polarization into the M1 subtype; therefore, this result could explain the dominance of the M1 subtype in this study [48].

It is noteworthy that the findings of the study are novel due to the young age of the patients and the severity of the defect, which made tissue acquisition highly complicated. Previous morphological studies using tissue samples have focused more on older children, specifically, children of an age for mixed dentition [23,24,49], or animal tissues [50,51,52].

The main limiting factor of the study could be the small sample size of both the control and patient groups, which was due to the previously described challenges of tissue acquisition, as well as the accessibility of samples from necropsies that fit all inclusion criteria.

Other limitations include lack of inter-rater reliability testing, which would be necessary to demonstrate the credibility of the presented results. We acknowledge that without this test, there is a potential for observer bias.

In addition, the control group presents a few limitations. Due to the challenges of acquiring samples from healthy controls, the control group presents fairly significant heterogeneity, which could lead to masked or misleading results and inaccurate conclusions. For this reason, it would be best to use a wider variety of control samples that vary more in the categories of age and sex.

Moreover, the reliability and reproducibility of the results should increase with the addition of effect size and confidence interval calculations, despite the fact that these calculations are mostly used in clinical studies and are seldom applied to the quantification of positive structures in preclinical research. Lastly, due to the suggestive nature of these correlations and their explanations, it would be beneficial to research the plausibility of these assumptions via pilot studies.

In order to gain clearer and more detailed information about the changes in these factors in clefted tissue and their relation to the morphopathogenesis of cleft lip and palate and the associated inflammatory conditions, it would be beneficial to analyze gene distribution by in situ hybridization and gene mapping and quantify factors by ELISA. The combined evaluation of singular factors by both IHC and gene-level examination could make the results more quantitative and unbiased and also broaden the understanding of particular changes in these factors and their possible causes.

Moreover, it would be extremely beneficial to correlate the changes in factor quantity with the clinical status and outcomes of the patients at specific points in time as they undergo many follow-up surgeries; such a study would connect observational morphological data with clinical data and challenges.

## 5. Conclusions

The tissues associated with unilateral cleft lip and palate presented significant increases in all of the tissue-associated immune and angiogenesis factors except M2 macrophages, suggesting the intensification of the above-mentioned processes in the tissue. Changes in total macrophage numbers and the amount of CD163 could illustrate an equilibrium shift, most likely towards M1 macrophages and pro-inflammatory activity.

The increase in VEGF, HSP60 and 70, NF-κB p50, and p65 could possibly be a counterbalancing and protective mechanism activated in response to the cellular stress created by the environment of chronic inflammation.

The absence of changes in TGF-β1 suggests a lack of connective-tissue involvement in the morphopathogenesis of cleft lip and palate.

The presence of mutual positive correlations indicates that are probably various synergistic and mutually initiating mechanisms, which could ensure a more stable and profound function of tissue-associated immune and angiogenesis factors.

## Figures and Tables

**Table 1:** Description of the patients.

Patient Number	Age (Months)	Sex	Family Anamnesis
1	3	M	
2	4	M	
3	4	M	Family history of epilepsy.
4	4	F	
5	4	F	
6	4	M	Mother had a kidney inflammation in the third gestational month.
7	4	F	
8	4	M	Mother had an inherited cleft.
9	4	F	Mother has varicose veins, lung insufficiency. Father has arrythmia.
10	5	M	Family history of Down’s syndrome.
11	5	M	Mother has had an echinococcus infection. Niece of mother’s mother has a child with a cleft.
12	5	F	
13	5	M	Mother has chronic sinusitis. Father smokes and has a history of surgically treated kidney tumor.
14	5	F	
15	8	M	Both parents smoke.

Abbreviations: M, male; F, female.

**Table 2:** Description of the control group [20].

Control Number	Age	Sex	Cause of Death
1a	Newborn	M	Birth asphyxia
2a	Newborn	F	Birth asphyxia
4a	24 weeks old	F	Abortion due to maternal indication
5a	Newborn	F	Sudden infant death syndrome
6a	Newborn	F	Sudden infant death syndrome

Abbreviations: M, male; F, female.

**Table 3:** Information about the antibodies used in IHC.

Tissue Factor	Product Code	Species	Working Dilution	Company	Location
VEGF	orb191500	Rabbit	1:100	Biorbyt Ltd.	Cambridge, UK
TGF-β1	orb77216	Rabbit	1:100	Biorbyt	Cambridge, UK
NF-κB p50	ab7971	Rabbit	1:100	Abcam	Cambridge, UK
NF-κB p65	orb37069	Rabbit	1:100	Biorbyt Ltd.	Cambridge, UK
HSP 60	sc-1052	Goat	1:1000	Santa Cruz Biotechnology, Inc.	Dallas, TX, USA
HSP 70	33-3800	Mouse	1:100	Invitrogen	Waltham, MA, USA
Macrophages	ab56297	Rat	1:100	Abcam	Cambridge, UK
CD 163	ab87099	Rabbit	1:200	Abcam	Cambridge, UK

Abbreviations: VEGF, vascular endothelial growth factor; TGF-β1, transforming growth factor beta 1; NF-κB, nuclear factor kappa B; HSP, heat-shock protein.

**Table 4:** Explanation of identifiers used in semi-quantitative evaluation [24,25].

Identifier Used	Explanation
0	No positive structures (0%)
0/+	Rare occurrence of positive structures (12.5%)
+	Few positive structures (25%)
+/++	Few to moderate numbers of positive structures (37.5%)
++	Moderate number of positive structures (50%)
++/+++	Moderate to numerous positive structures (62.5%)
+++	Numerous positive structures (75%)
+++/++++	Numerous to abundant positive structures (87.5%)
++++	Abundance of positive structures (100%)

**Table 5:** Semi-quantitative assessment of VEGF, TGF-β1, NF-κB p105/p50, and NF-κB p65 in the patient and control groups.

Sample Number	VEGF			TGF-β1			NF-κB p105/p50			NF-κB p65		
	EP	CT	BV	EP	CT	BV	EP	CT	BV	EP	CT	BV
1	+++	++/+++	+++	+/++	+	+/++	++	+	++	++	0/+	+
2	++	0/+	+	+	+/++	0/+	+++	++	++	+++	+/++	++/+++
3	0/+	++/+++	+/++	+	+/++	0/+	++	+	+	+++/++++	+	+++
4	++	0/+	+++	+	+	0/+	+++	+	+++	+++	+	+++
5	+	0/+	0/+	+	+/++	+	++/+++	+/++	+	+++	++	++
6	++/+++	+	0/+	+/++	++	+	+++	++	++	+++	++	+++
7	0	0	0	+/++	+	0	+++	++	+	+++/++++	+	++
8	+/++	++/+++	+++	++	++	+	++	++	++	+++	+/++	+++
9	+	0/+	+	++	+/++	+	++	++	++	+++	0/+	++
10	++/+++	0/+	0/+	+	0/+	0	++	0	+	+++	0/+	+
11	++	+	++	+	0/+	0/+	++/+++	+	++	+++	0/+	+
12	++	0/+	++	+	++	+	++	+/++	++	+++	+	+++
13	0/+	0/+	0	++	++	+	+++	++	+	+++	++	++/+++
14	0/+	+	+/++	0/+	+	+	++/+++	++	++	+++	++	+++
15	+++	0/+	+	+/++	0/+	+	++/+++	+	+	++/+++	+	++
**Patient Median**	++	0/+	+	+	+/++	+	++/+++	+/++	++	+++	+	++
1a	0	+	+	+	+	+	++	++	+++	+	+	+
2a	0	++	+	++	++	+	+	+++	+++	+/++	+/++	+
3a	+	+	+	++	++	++	+++	++	+++	+/++	+/++	+
4a	0	+	0	+	+	+	+	+	+	+	+	0
5a	0	++	+	0	+	+	+	++	+	0	0/+	0
**Control Median**	0	+	+	+	+	+	+	++	+++	+	+	+

Abbreviations: EP, epithelium; CT, connective tissue; BV, blood vessels; VEGF, vascular endothelial growth factor; TGF-β1, transforming growth factor beta 1; NF-κB, nuclear factor kappa B. The gray back color highlights the median values for each of the factors.

**Table 6:** Median values for VEGF, TGF-β1, NF-κB p105/p50, and NF-κB p65 in the patient and control groups.

Sample Number	VEGF			TGF-β1			NF-κB p50			NF-κB p65		
	EP	CT	BV	EP	CT	BV	EP	CT	BV	EP	CT	BV
P	++	0/+	+	+	+/++	+	++/+++	+/++	++	+++	+	++
C	0	+	+	+	+	+	+	++	+++	+	+	+
U-test value	7.307	2.722	0.887	0.019	0.101	3.004	4.004	2.156	1.267	12.873	0.051	8.842
*p*-value	0.007	0.099	0.346	0.889	0.750	0.083	0.045	0.142	0.260	0.000	0.821	0.003

Abbreviations: EP, epithelium; CT, connective tissue; BV, blood vessels; VEGF, vascular endothelial growth factor; TGF-β1, transforming growth factor beta 1; NF-κB, nuclear factor kappa B.

**Table 7:** Semi-quantitative assessment of HSP 60, HSP 70, macrophages, and CD 163 in the patient and control groups.

Sample Number	HSP 60			HSP 70			Macrophages			CD 163		
	EP	CT	BV	EP	CT	BV	EP	CT	BV	EP	CT	BV
1	+++	+	0	+/++	0	0	++	+	+	0/+	0/+	0/+
2	+++	+	+	+	0	0	+++	+	+++	0	0/+	0/+
3	+/++	+	0	+/++	0	0/+	+++	++	+++	0	0/+	0/+
4	+++	+	+	+/++	0	0	+++	+	++	0	+	+
5	++/+++	+	+	+	+	+	+++	++	++	0	+/++	+
6	+++	++	0/+	++/++	+	0	++/+++	++	++/+++	+	+	0/+
7	++++	++	+++	++	+	+	+++	+	++	0	+/++	+
8	++	0	0	++	0	0	+	+	+	0	++	0
9	+++	0	0	0/+	0	0/+	++	0/+	0/+	0	+	0/+
10	++	0	0/+	++	0	0	+++/++++	++	++	0	+/++	0/+
11	++	0	0	+	+	0	+	0	0	0	0/+	0/+
12	++/+++	0	++	+/++	0	0/+	+++	+	+++	0	+	+
13	++	0/+	0	++	0	0/+	+++	+++	++	0	+	+
14	+++	0/+	+	++	+	0	++	+++	+	0	+	0/+
15	++	0/+	0/+	++	0	0	+/++	++	++	0	+/++	+
**Patient Median**	++/+++	0/+	0/+	+/++	0	0	+++	+	++	0	+	0/+
1a	0	0	0	0	0	0	++	++	+++	+	+	+
2a	0	0	0	0	0/+	0	+	+	+++	+	++	+
3a	+	+	0	0	0	0/+	0	0	+	++	++	++
4a	+	+	0	0	0	0/+	+	+	+	0	+	+
5a	0	0	0	0	0	0	+	++	+	0	+	+
**Control Median**	0	0	0	0	0	0	+	+	+	+	+	+

Abbreviations: EP-epithelium; CT-connective tissue; BV-blood vessels; HSP-heat-shock protein. The gray back color highlights the median values for each of the factors.

**Table 8:** Median values for HSP 60, HSP 70, macrophages and CD 163 in patient and control groups.

Sample Number	HSP 60			HSP 70			Macrophages			CD 163		
	EP	CT	BV	EP	CT	BV	EP	CT	BV	EP	CT	BV
P	++/+++	0/+	0/+	+/++	0	0	+++	+	++	0	+	0/+
C	0	0	0	0	0	0	+	+	+	+	+	+
U-test value	11.229	0.693	1.867	11.301	0.667	0.040	7.331	0.307	0	5.030	1.335	6.095
*p*-value	0.001	0.405	0.172	0.001	0.414	0.841	0.007	0.579	1	0.025	0.248	0.014

Abbreviations: EP-epithelium; CT-connective tissue; BV-blood vessels; HSP-heat-shock protein.

## Data Availability

All datasets used in the present study are available in the Section 3.

## Abbreviations

The following abbreviations are used in this manuscript:

M	Multidisciplinary Digital Publishing Institute
F	Directory of open access journals
VEGF	Vascular endothelial growth factor
TGF-β1	Transforming growth factor beta 1
NF-κB	Nuclear factor kappa B
HSP	Heat shock protein
EP	Epithelium
CT	Connective tissue
BV	Blood vessels
